# Structural Characterization of a Novel Pectin Polysaccharide from Mango (*Mangifera indica* L.) Peel and Its Regulatory Effects on the Gut Microbiota in High-Fat Diet-Induced Obese Mice

**DOI:** 10.3390/foods14162910

**Published:** 2025-08-21

**Authors:** Ruyan Fan, Wenting Zhang, Lang Wang, Tao Fei, Jianbo Xiao, Lu Wang

**Affiliations:** 1 School of Food Science and Engineering, Hainan University, Haikou 570228, China; 23210832000022@hainanu.edu.cn (R.F.); floriazwt@163.com (W.Z.); inwanglang@outlook.com (L.W.); mighty0223@163.com (T.F.); 2 Key Laboratory of Food Nutrition and Functional Food of Hainan Province, Hainan University, Haikou 570228, China; 3 Nutrition and Bromatology Group, Department of Analytical and Food Chemistry, Faculty of Sciences, University of Vigo, 32004 Ourense, Spain

**Keywords:** pectin polysaccharide, structural characterization, obesity, SCFAs, gut microbiota

## Abstract

The gut microbiota plays a significant role in metabolic diseases such as obesity. We extracted and purified a new type of pectin polysaccharide (mango peel pectin, MPP) from mango (*Mangifera indica* L.) peel. The structural analysis results reveal that MPP has a molecular weight (Mw) of 6.76 × 10^5^ Da and the mass fractions of the main components were galacturonic acid (21.36%), glucose (8.85%), and arabinose (5.97%). The results of methylation and NMR analyses reveal that the backbone of MPP consisted of →6)-α-D-GalpAOMe-(1→ and →4)-*β*-D-Glcp-(1→ linkages. Based on the above structural analysis, we further explored the therapeutic effect of MPP on high-fat diet-induced obese mice. The results demonstrate that MPP significantly suppressed body weight and dyslipidemia, reduced liver damage and lipid accumulation, attenuated changes in adipocyte hypertrophy, and improved glucose homeostasis and insulin resistance, with fasting blood glucose (FBG) levels decreasing by more than 12.8%. Furthermore, the modulatory impact of MPP on gut microbiota composition was investigated. MPP treatment significantly enhanced the levels of short-chain fatty acids (SCFAs) by decreasing the amount of Bacillota and reducing the Bacillota/Bacteroidota ratio, especially with an increase in the total SCFA content of over 64%. Meanwhile, MPP treatment encouraged beneficial bacteria to grow (e.g., Bacteroidota, *Akkermansia*, and *Nanasyncoccus*), altered the gut microbiome profiles in mice, and decreased the abundance of harmful bacteria (e.g., *Paralachnospira*, *Coproplasma*, *Pseudoflavonifractor*, *Parabacteroides*, *Acetatifactor,* and *Phocaeicola*). Overall, the findings demonstrate for the first time that MPP treats obesity by alleviating dyslipidemia, improving insulin resistance, and regulating gut microbiota to improve the intestinal environment.

## 1. Introduction

At present, obesity has become a critical burden across developed and developing nations. This chronic metabolic disorder disrupts normal physiological processes and acts as a precursor to multiple comorbidities, such as type 2 diabetes, cardiovascular complications, elevated blood pressure, and dyslipidemia [1,2]. Recent epidemiological projections estimate that over 1 billion individuals globally have developed obesity since 1990 (WHO, 2023), while contemporary modeling predicts this figure may reach 4 billion individuals with excessive body weight by 2035 [3] (World Obesity Federation, 2023). Accumulating evidence has established a bidirectional relationship between gut microbial dysbiosis and obesity pathogenesis [4,5,6]. Therefore, regulating the gut microbiota to prevent or alleviate obesity has emerged as a promising novel approach.

Influenced by a dynamic interplay of environment, diet, and genetics, research on the gut microbiota is rapidly advancing [7]. The microbiota is primarily composed of the phyla Bacteroidota and Bacillota [8]. Pioneering studies in microbial ecology have identified enrichment of Bacillota as a pro-obesogenic signature, while depletion of Bacteroidota correlates with disrupted lipid homeostasis in metabolic disorders [9]. In addition, an elevated Bacillota/Bacteroidota ratio is closely related to obesity symptoms [10]. To date, numerous studies have confirmed that modulating the gut microbiota benefits obesity management. The gut microbiota influences human metabolism and disease through metabolites such as short-chain fatty acids (SCFAs) and bile acids [11]. Zhou et al. demonstrated that Fuzhuan tea (FBT) can effectively increase the abundance of *Akkermansia* muciniphila, *Alloprevotella*, *Bacteroides*, and *Faecalibaculum*, thereby alleviating obesity [12]. Xiong et al. demonstrated that reduced gut microbiota diversity was associated with increased adiposity, dyslipidemia, impaired glucose homeostasis, and insulin resistance in obese humans [13]. Therefore, regulating the gut microbiota represents a promising therapeutic strategy for obesity.

Recent studies have extensively investigated the potential therapeutic applications of pectin in managing obesity and its associated metabolic disorders. Zhao et al. demonstrated that apple pectin-enriched obesity-related bacteria (*Coriobacteriaceae*), pectin-degrading bacteria (*Faecalibaculum*), and *Ruminococcus* reduced propionic acid levels, collectively alleviating colon damage and hepatic steatosis [5]. Research has found that, by regulating lipid metabolism and the immune system, the mechanism by which polysaccharides improve obesity can be elucidated [14]. Pectin derived from *Premna microphylla* Turcz leaves (PTP) exhibits anti-obesity properties by suppressing adiposity in experimental models, reducing visceral fat, and decreasing the abundance of harmful bacteria, thereby effectively alleviating obesity symptoms [15]. Zhang et al. confirmed that mangiferin protected neurological function and relieved post-stroke cognitive impairment by improving lipid metabolism abnormalities in ischemia/reperfusion rats [16]. Mango peel contains substantial quantities of cell wall-derived polysaccharides (pectin, cellulose) and monomeric units (e.g., arabinose, galactose, mannose), with these bioactive compounds constituting approximately 15–25% of the fruit’s dry mass and representing underutilized bioresources in mango industrial processing [17]. However, as a byproduct of processing, mango peel is discarded in large quantities, contributing to environmental pollution. In addition, the impact of mango peel pectin on gut microbiota in obesity remains incompletely studied.

In this study, we attempted to structurally characterize mango peel pectin (MPP) as well as to explore the significance of MPP in the gut microbiota of obese mice. Firstly, we isolated and purified MPP and identified its structure. Secondly, we used obese mice as a model to investigate the impact of different MPP doses. By assessing indicators such as lipid levels, liver and adipose tissue changes, glucose homeostasis, and insulin resistance, as well as changes in intestinal flora and SCFAs, we explored the regulation of intestinal flora by MPP for the purpose of alleviating obesity. This study provides a promising theoretical foundation for developing functional pectin-based interventions.

## 2. Materials and Methods

### 2.1. Reagents

Triglyceride (TG), total cholesterol (TC), low-density lipoprotein cholesterol (LDL-C), high-density lipoprotein cholesterol (HDL-C), aspartate transaminase (AST), and alanine aminotransferase (ALT) were purchased from Nanjing Jiancheng Biological Engineering Research Institute (Nanjing, China). The E.Z.N.A.^®^ Stool DNA Kit was purchased from Omega Bio-tek (Norcross, GA, USA). Jiangsu Synergy Pharmaceutical and Biological Engineering Co. (Nanjing, China) provided the irradiated daily maintenance feed and irradiated high-fat feed (18.14% protein, 60.65% fat, and 21.22% carbohydrate) required by the animals.

### 2.2. Extraction and Purification of MPP

**Figure 1:**
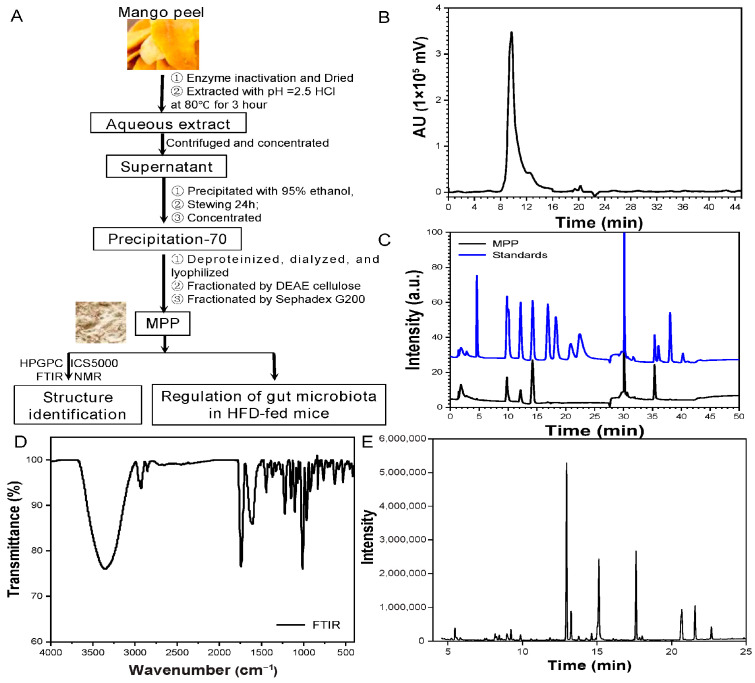
Extraction and purification of pectin and structural analysis of MPP. Mango peel gum processing flow chart (**A**). Molecular weight analysis of MPP (**B**). Monosaccharide composition analysis of MPP (**C**). FTIR analysis of MPP (**D**). The total ion diagram for MPP methylation analysis (**E**).

Based on a previous pectin method of extraction, pectin was extracted and purified from mango peel procured from the local market using the process shown in Figure 1A [18]. Firstly, mango peel was immersed in boiling water for enzyme inactivation, and then dried at 60 °C to obtain mango peel powder. The dried mango peel was ground using a Baihaojia grinder, followed by sieving through an 80-mesh standard sieve. The powder (10 g) was subjected to pectin extraction using HCl (pH = 2.5) at 80 °C for 3 h. After centrifugation for 10 min, clarified fractions were precipitated with 95% ethanol. Finally, the precipitate was concentrated to obtain crude pectin. To purify the pectin, proteins and pigments were removed using D301-R macroporous resin, followed by dialysis using dialysis membranes with a retention rate of 5 kDa. Purified mango peel pectin (MPP) was obtained via sequential purification using DEAE-cellulose ion-exchange chromatography followed by Sephadex G-200 size-exclusion chromatography, with real-time polysaccharide monitoring through an online refractive index detector (RID) and quantitative validation via the phenol-sulfuric acid assay using a Thermo Scientific Multiskan GO microplate reader (Thermo Fisher Scientific, Waltham, MA, USA). MPP was freeze-dried and stored at −20 °C for later use.

### 2.3. Structural Analysis of MPP

#### 2.3.1. Molecular Weight Analysis of MPP

The molecular weight (Mw) of MPP was determined by high-performance gel-permeation chromatography (HPGPC; Waters 1515 system, Milford, MA, USA). During molecular weight determination, retention times were monitored using a refractive index detector (RID). Referring to previous studies, a dextran standard series was used to construct the calibration curve [19]. MPP was dissolved (0.1 M NaNO_3_) and filtered using 0.45 μm cellulose membrane. Ultrapure water was chosen as the mobile phase for elution at a flow rate of 1 mL/min. The temperature of the column was kept constant at 30 °C. A calibration curve was established by linear regression using retention time and molecular weight of dextran standards to calculate the Mw of MPP.

#### 2.3.2. Monosaccharide Composition Analysis of MPP

The monosaccharide composition of MPP was analyzed using a chromatography instrument (Thermo Fisher Scientific, Waltham, MA, USA). The analysis method was modified from the previous method [20]. Samples were hydrolyzed with 2 M trifluoroacetic acid (TFA) at 121 °C for 2 h, dried under nitrogen stream and washed thrice with methanol to remove residual TFA, and then reconstituted in ultrapure water for analysis. Samples were dissolved and were filtered using a 0.45 μm nylon membrane before being separated using a CarboPac™ PA20 analytical column (Dionex™, Thermo Fisher Scientific, Waltham, MA, USA). The mobile phase consisted of deionized water (Phase A), 0.1 M NaOH solution (Phase B), and a mixture of 0.1 M NaOH solution and 0.2 M NaAc (Phase C). Chromatographic separation was conducted under isocratic elution conditions with a 0.5 mL/min eluent flow rate coupled to thermostatically controlled column stabilization at 30 °C. A calibration curve was linearly fitted to the retention time and standard response values for the determination of MPP monosaccharide fractions.

#### 2.3.3. FTIR Analysis

The powder was milled together with potassium bromide (KBr, 1:100) and pressed into a pellet. The Fourier Transform Infrared (FTIR) spectrum of the sample was recorded using a Bruker T27 spectrometer (Germany) in the 400–4000 cm^−1^ wavenumber range. 

#### 2.3.4. Methylation Analysis

In order to measure the degree of MPP methylation as well as the finer structure, MPP was methylated and then analyzed using gas chromatography—mass spectrometry (GC-MS, Agilent 7890A-5977B, Agilent Technologies Inc., Santa Clara, CA, UAS). The sample was dissolved in ultrapure water, derivatized with 1-cyclohexyl-3-(2-morpholinoethyl) carbodiimide methyl p-toluenesulfonate, and divided into two aliquots with 2 M imidazole. Each aliquot was reduced with NaBH_4_ or NaBD_4_, lyophilized, reconstituted in DMSO, and alkylated with NaOH. Methylation was performed with iodomethane. The product was extracted with dichloromethane (DCM), dried under N_2_, and purified via sequential imidazole treatments and triple DCM washes. Residual reagents were quenched with NH_4_OH and NaBD_4_ before the GC-MS analysis of the DCM phase. We performed the GC-MS chromatographic analysis using a BPX70 column. The initial temperature of 140 °C was held for two min and then ramped to 230 °C at 3 °C/min for three min [21].

#### 2.3.5. NMR Analysis

In order to further elucidate the structural features of MPP, nuclear magnetic resonance (NMR) spectroscopy was performed. The pectin solution was first prepared and then filled into the NMR tube. For NMR analysis, purified polysaccharide was dissolved in D_2_O (≥40 mg/mL), transferred to a 5 mm NMR tube (0.5 mL volume), and analyzed using a Bruker Avance III HD 600 MHz spectrometer. Conventional ^1^H and ^13^C NMR spectra were collected through basic pulse sequences. Comprehensive structural characterization was achieved through multidimensional NMR analyses, including correlation spectroscopy (COSY), heteronuclear single-quantum coherence (HSQC), heteronuclear multiple-bond correlation (HMBC), and nuclear Overhauser effect spectroscopy (NOESY) experiments, all conducted with parameter optimized acquisition protocols. All spectra were recorded at 25 °C [22].

### 2.4. Animal Experiments Design

A cohort of 50 male mice (body weight of 20 ± 2 g) obtained from Vital River Laboratory Animal Technology Co. (Beijing, China) was acclimatized in a specific pathogen-free (SPF) facility under controlled conditions: ambient temperature maintained at 22 °C and a 12-h light/dark photoperiod. The Animal Care Committee of Hainan University approved the animal experiments in this study (HNUAUCC-2023-00199). Fifty mice were randomly divided into five groups (*n* = 10) after one week of adaptive feeding: the ND group (normal diet and saline solution), the MPP group (normal diet and MPP 200 mg/kg BW), the HFD group (high-fat diet and saline solution), the MPPL group (high-fat diet and MPP 100 mg/kg BW), and the MPPH group (high-fat diet and MPP 200 mg/kg BW). The corresponding physiological saline and MPP were administered daily, and the food and water intake of mice was observed every two days. The weight of mice was measured once a week and recorded. After a 9-week MPP intervention period, experimental mice underwent anesthesia for biological sample collection. Ocular venous blood samples were acquired and promptly subjected to 15 min centrifugation at 4 °C for serum isolation. Hepatic and white adipose tissues (WATs) were excised and weighed to determine organ indices through the following formula: (tissue mass/body weight) × 100%. Representative tissue sections were fixed in 4% paraformaldehyde solution for subsequent histological examinations, including hematoxylin and eosin (H & E) staining and Oil Red O lipid visualization. Intestinal contents from the colonic region were snap-frozen at −80 °C for future microbiota characterization and short-chain fatty acid (SCFA) quantification.

### 2.5. Glucose Homeostasis

Three days before the end of the experiment, all fasted mice (after a 12-h food restriction) underwent an oral glucose tolerance test (OGTT) by administering a 2 g/kg glucose solution via oral gavage. Blood glucose concentrations were measured at 0 min, 30 min, 60 min, 90 min, and 120 min intervals using the Accu-Chek Performa monitoring system (Mannheim, Germany). The glucose response curve was analyzed using trapezoidal integration to determine the total area under the curve (AUC). Insulin resistance was quantified using the homeostasis model assessment (HOMA-IR) through the following equation: (Fasting Blood Glucose × Fasting Insulin)/22.5.

### 2.6. Biochemical Parameter Analysis

Absolute alcohol or normal saline was added to the liver, which was then homogenized using a high-throughput tissue grinder (KZ-III-F, Servicebio, Wuhan, China) to obtain the supernatant. The levels of TC, TG, LDL-C, and HDL-C in serum, as well as the activities of AST and ALT in liver tissue, were measured using reagent kits.

### 2.7. Histological Analysis

Adipose tissue was fixed, embedded in paraffin, sectioned, and stained with H & E. Liver samples were stained with Oil Red O. Histological changes in adipose and liver tissues were observed using an optical microscope (Eclipse Ci-L, Tokyo, Japan) and images were captured at 400× magnification.

### 2.8. Metagenomic Sequencing

Microbial DNA was extracted from the intestinal contents of the mice samples using the E.Z.N.A.^®^ stool DNA Kit (Omega Bio-tek, Norcross, GA, USA), according to the instruction manual. Metagenomicshotgun sequencing libraries were constructed and sequenced at Shanghai Biozeron Biological Technology Co., Ltd. (Shanghai, China). α-diversity, β-diversity, principal component analysis (PCA), and gut microbiota composition analyses were performed on the sequencing results. In briefly, for each sample, 1 μg of genomic DNA was sheared by Covaris S220 Focused-ultrasonicator (Woburn, MA, USA), and sequencing libraries were prepared with a fragment length of approximately 450 bp. All samples were sequenced in the Illumina NovaSeq 6000 instrument in the pair-end 150 bp (PE150) mode.

### 2.9. Analysis of SCFAs

Samples were homogenized in 80% methanol, followed by protein precipitation via centrifugation (12,000 rpm, 10 min). SCFAs were derivatized with 150 μL of derivatization reagent (3-nitrophenylhydrazine) at 40 °C for 40 min after protein precipitation (80% methanol, 12,000 rpm × 10 min). The internal standard solution was a mixture of isotope-labeled SCFA standards stored at −20 °C, with 5 μL added to 95 μL of derivatized supernatant prior to LC-MS/MS injection. Chromatographic separation was performed using a Waters ACQUITY UPLC BEH C18 column (2.1 × 100 mm, 1.7 μm) with a binary mobile-phase system comprising ammonium a glycosidic acetate aqueous solution and acetonitrile–isopropanol mixture. The mobile phase consisted of solvent A (10 mM ammonium acetate in water) and solvent B (acetonitrile: isopropanol, 1:1). Quantification of SCFAs was performed using isotope dilution mass spectrometry, with matrix-matched calibration standards prepared by serial dilution of SCFA stock solutions in an analyte-free matrix. The results were normalized to sample mass and reported as mg/g.

### 2.10. Statistical Analysis

Statistical analyses were conducted using the IBM SPSS Statistics 27 software, with a one-way ANOVA followed by Duncan’s multiple comparisons. Statistical significance was assessed at a threshold of *p* < 0.05, and data are presented as mean ± standard deviation (SD).

## 3. Results and Discussion

### 3.1. Subsection Extraction, Purification, and Identification of MPP

Pectin is a natural food polysaccharide known for its nutritional and functional properties that has been widely studied. It is a high Mw composite carbohydrate found in the cell walls of various plants [23,24,25]. Pectin has previously been shown to be beneficial in alleviating HFD-induced obesity-like metabolic disorders and in alleviating related disorders such as hyperlipidemia and dyslipidemia [26]. Pectin effectively helps to produce a layer of mucus that protects intestinal epithelial cells from becoming damaged [27]. In addition, pectin is fermented and broken down by intestinal microorganisms and produces SCFAs, which beneficially affect the metabolism and body health. In particular, Bacteroide and *Prevotella* in the gut can degrade pectin, and then, pectin fermentation products can promote the development of beneficial bacteria, thus improving organismal health [28,29]. The results of the structural characterization analysis of pectin are presented below [30].

The HPGPC results of MPP are shown in Figure 1B. The Mn, Mp, Mw, and Mz of MPP are 4.97 × 10^5^, 6.12 × 10^5^, 6.76 × 10^5^, and 8.60 × 10^5^, respectively. Different process parameters have varying effects on the extraction and purification of pectin. By comparing with the literature, we found that the precise control of extraction and purification conditions adopted in this study was the key to balancing yield and structure, as well as the key to obtaining a high molecular weight in MPP [31]. Dispersity (DM), expressed as the weight average molecular weight divided by the number average molecular weight, reflects the breadth of the molecular weight distribution of the polymer. A lower DM value corresponds to a more uniform molecular weight profile, while a value of 1 signifies a perfectly monodisperse polymer system where all chains possess identical lengths [32,33,34]. The dispersion coefficient (Mw/Mn) of MPP was 1.36, and the HPGPC results show a relatively clear peak, indicating that the Mw distribution of MPP is relatively narrow and uniform.

Further component studies were carried out on MPP, and the ion exchange chromatography results are presented in Figure 1C. The elution order of the reference materials corresponds to their increasing retention times observed in the chromatographic analysis; they were fucose (4.6 min), arabinose (9.81 min), rhamnose (10.07 min), galactose (12.17 min), glucose (14.26 min), xylose (16.89 min), mannose (18.28 min), fructose (20.78 min), galacturonic acid (35.39 min), and glucuronic acid (38.09 min). The chromatographic results show that MPP was made up mainly of galacturonic acid, glucose, and arabinose, with contents (μg/mg) of 213.59, 88.46, and 59.71, respectively.

As shown in Figure 1D, FTIR provided an analytical platform for the structural characterization of pectin polysaccharides, where characteristic absorbance bands correlate with specific molecular vibrations indicative of functional group fingerprints. A broad strong peak at 3379 cm^−1^ belongs to the OH- stretching vibrational group [35]. The faint absorption observed at 2973 cm^−1^ originates from the C-H stretching modes in methyl, methylene, and methine groups within the pectin polysaccharide backbone [36]. The infrared spectrum of pectin exhibits two characteristic absorption bands at 1750 cm^−1^ and 1631 cm^−1^, which correspond to the stretching vibrations of carbonyl-containing functional groups. These spectral features are specifically assigned to the C = O stretching modes of ester groups (-COOCH_3_) and the asymmetric stretching vibrations of carboxylate anions (-COO^−^), reflecting the structural motifs in the pectin polymer [37]. The weak absorption band observed at 1472 cm^−1^ can be assigned to the scissoring deformation vibrations of C-H bonds in methyl (-CH_3_) and methylene (-CH_2_-) groups, which corresponds to characteristic bending modes of aliphatic hydrocarbon chains in the pectin structure. This spectral feature further corroborates the presence of methoxy substituents (-OCH_3_) and hydrocarbon backbone components within the polymer system [38]. The C-O-C stretching vibration of the pectin polymer chain structure is responsible for the peak at 1213 cm^−1^ [39]. Neutral arabinosylated glycans of RG-I are associated with the peak at 1061 cm^−1^. In addition, arabinose, galactose, and rhamnose are mainly related to rhamnogalacturonan-I (RG-I), while xylose and galactose are mainly related to rhamnogalacturonan-II (RG-II) [40]. Based on the monosaccharide composition analysis above, it can be concluded that MPP contains small amounts of other polysaccharides.

The GC-MS results show that MPP contained mainly 11 identical derivatives (Table 1). Figure 1E shows the total ion diagram for MPP methylation analysis. The results show that the glycosidic linkage of MPP was dominated by (1→4)-linked galacturonic acid (4-Gal(p)-UA, 32.13%) and glucose (4-Glc(p), 22.24%) as the main chain, as well as Terminal-linking arabinose (t-Ara(f), 9.92%) and galacturonic acid (t-Gal(p)-UA, 8.93%) and (1→4)-linked galacturonic acid (4-Gal(p), 8.08%) in the form of branches, with small amounts of 5-Ara(f) (5.18%) and 6-Gal(p) (4.12%), in agreement with the results of the analysis of monosaccharides.

**Figure 2:**
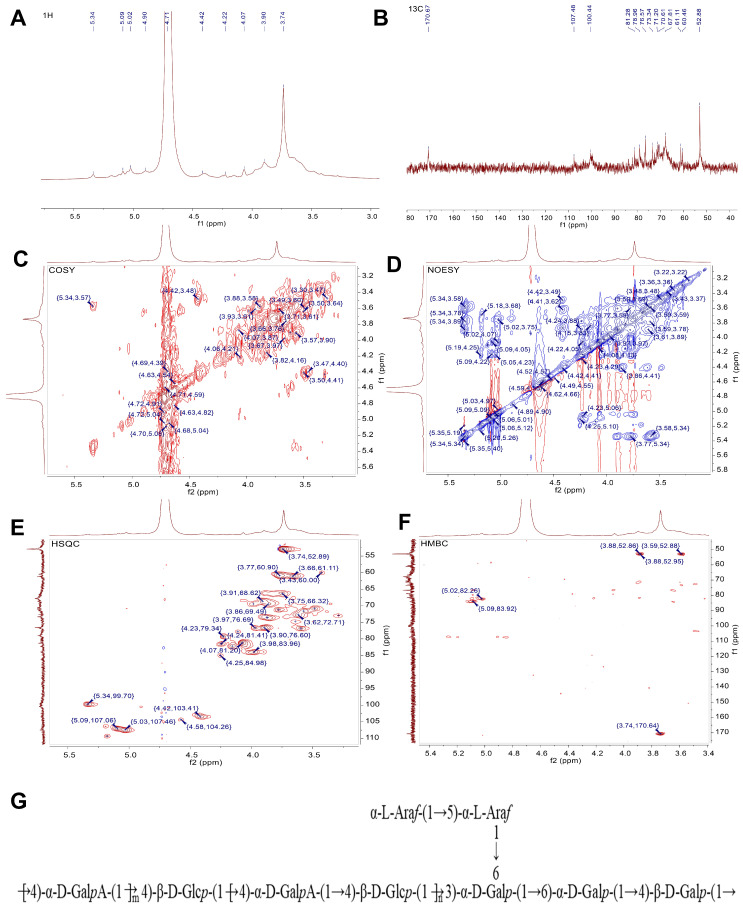
Nuclear magnetic resonance (NMR) analysis and structural repeating units of MPP. One-dimensional NMR spectroscopy of MPP—1H NMR (**A**). One-dimensional NMR spectroscopy of MPP—13C NMR (**B**). Two-dimensional NMR spectroscopy of MPP (**C**–**F**). Structure unit diagram of MPP (**G**).

One-dimensional proton NMR spectroscopy (^1^H NMR) serves as a critical tool for analyzing glycosidic linkage conformations in polysaccharides. Characteristic proton resonances in the 3.0–6.0 ppm range correspond to saccharide proton environments, with anomeric protons showing distinct chemical shifts: β-linkages generally appear at δ 4.3–4.8 ppm, while α-linkages predominantly resonate in the δ 4.8–5.8 ppm region [41]. On the basis of methylation analysis, heteronuclear correlation signals, and literature data, it is presumed that the sugar residues are D-galacturonic acid (D-GalA), D-galactose (D-Gal), D-glucose (D-Glu), and L-arabinose (L-Ara), as evidenced by the characteristic resonances of their anomeric carbon atoms [42,43]. Combined with the monosaccharide composition and methylation results, MPP is mainly composed of D-Glu and D-GalA. To analyze the possible structures and linkages in this polysaccharide, the ^13^C and ^1^H chemical shifts of each sugar residue of MPP were combined with the HMBC and NOESY spectra (Table 2 and Figure 2A–F). The schematic diagram of MPP structural unit repetition is shown in Figure 2G. It was deduced that the main chain of this polysaccharide mainly consisted of →4)-α-D-GalpA-(1→ and →4)-β-D-Glcp-(1→ and that the branched chain mainly consisted of α-L-Araf-(1→5)-α-L-Araf-(1→ attached to the O-6 position of sugar residue →3,6)-α-D-Galp-(1→), which is consistent with previous results [37]. The linkage form and conformation of the MPP glycosidic bonds were further explored by GC-MS and proton NMR experiments, and the final results confirm that MPP is mainly composed of D-GalA and D-Glu. Generally, pectin with a molecular weight below 100 kDa belongs to low-molecular-weight pectin, while pectin with a molecular weight higher than 180 kDa belongs to high-molecular-weight pectin. The pectin extracted in this experiment has a high molecular weight, which indicates greater stability and potentially enhanced bioactivity [44]. In addition, the process used to obtain MPP (resulting in a dispersity of 1.36) is simpler and more efficient compared to other extraction methods [45]. More significantly, there is currently no literature reporting the structural characteristics of mango peel pectin polysaccharides. The conformation and composition of MPP lay the foundation for the subsequent investigation of the mechanisms related to glycolipid metabolism and obesity.

### 3.2. MPP Prevented Obesity and Hepatic Steatosis in HFD-Fed Mice

Evidence suggests that prolonged intake of HFD could lead to liver dysfunction, metabolic imbalances in carbohydrate and lipid metabolism, and gastrointestinal flora disturbances, ultimately resulting in a persistent metabolic disorder [46]. As shown in Supplementary Materials Table S1, the body weight, food intake, water consumption, and organ-to-body weight ratios of mice were systematically monitored and analyzed during the 9-week feeding period. Measurements revealed no statistically significant differences (*p* > 0.05) among the five experimental groups. Notably, HFD-fed mice exhibited a marked increase in body weight compared to the ND controls (*p* < 0.01), thereby validating the successful establishment of the diet-induced obesity model. Furthermore, administration of MPP significantly attenuated weight gain across all three MPP-treated groups and in a dose-dependent manner (*p* < 0.05 compared with the HFD group), aligning with prior mechanistic investigations demonstrating the anti-obesity effects of polysaccharide derivatives through the modulation of lipid metabolism pathways [47]. Longitudinal monitoring revealed no statistically significant intergroup differences in food intake or water consumption profiles throughout the 9-week regimen, confirming that metabolic changes were independent of feeding behavior alterations. Therapeutic intervention with MPP dose-dependently reversed these pathological alterations. The anti-obesity efficacy of MPP is mechanistically attributable to its unique polygalacturonic acid backbone, as characterized by ^1^H NMR spectroscopy. This structural configuration enhances viscous fiber properties, enabling formation of gastric hydrogel complexes that delay nutrient absorption and modulate PPAR-γ-mediated adipogenesis, consistent with established mechanisms of pectin derivatives in metabolic regulation [48]. Pectin exhibits a high hydration capacity; upon contact with water, it rapidly absorbs water and, in vivo, prolongs gastric emptying. Furthermore, it increases the secretion of hormones, which are produced when gut microorganisms ferment pectin to generate acetic acid and propionic acid. This process inhibits food interaction with intestinal enzymes, facilitating intestinal transport and ultimately reducing fat accumulation to exert a body-weight-lowering effect [49,50].

**Figure 3:**
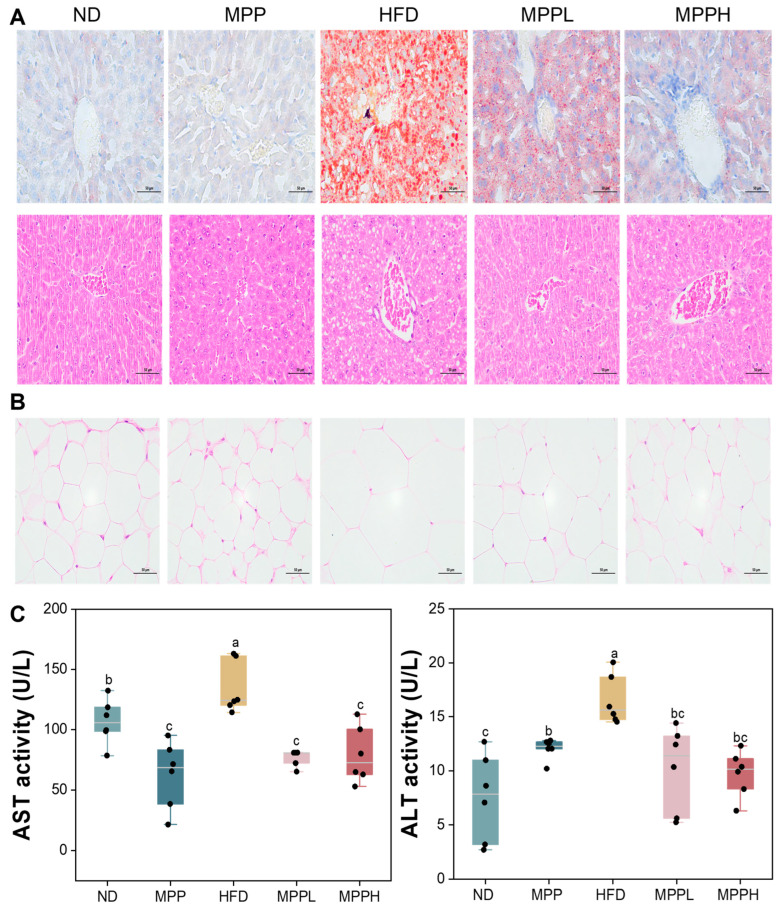
The effect of MPP intervention on liver and adipocytes in obese mice. Representative results of liver tissue oil red and H & E staining (400×) (**A**). Representative results of oil red staining of adipose tissue (400×) (**B**). Indicators related to liver injury, AST and ALT (**C**). ND, MPP, HFD, MPPL, and MPPH are the names of the five experimental groups in this study, representing, respectively, normal diet and saline solution, normal diet and MPP 200 mg/kg BW, high-fat diet and saline solution, high-fat diet and MPP 100 mg/kg BW, and high-fat diet and MPP 200 mg/kg BW. Data are mean ± SD (*n* = 6). Data with different letters indicate significantly differences (*p* < 0.05).

The main metabolic and endocrine organs associated with obesity are adipose tissue and the liver [51]. Adipocytes are the units of adipose tissue and store lipids, and the amount of stored lipid directly influences adipocyte size [52]. To further confirm the health benefits of MPP in obese mice, histological observations of liver and adipose tissue were carried out after the end of the experiment. As shown in Figure 3A, liver tissue in the HFD group had significant deformation, vacuolization, and abnormal adipose tissue accumulation, but the MPP treatment attenuated these alterations. In addition, the diameter of adipocytes was also abnormally increased in the HFD group, which was ameliorated by MPP treatment, as shown in Figure 3B. Notably, the improvement in adipocyte size was dose-dependent, and even in the regular feed group, adipocyte diameters in the MPP-treated mice were reduced compared to those in the ND group. Moreover, AST and ALT are important indicators to judge liver damage. As shown in Figure 3C, the hepatic biomarkers AST and ALT exhibited marked elevation in the HFD group relative to the ND group (*p* < 0.05), while the MPP-administered groups demonstrated comparable levels without statistical significance. In summary, MPP has health benefits in alleviating HFD-induced obesity.

### 3.3. MPP Alleviated Abnormal Glucolipid Metabolism in HFD-Induced Obese Mice

**Figure 4:**
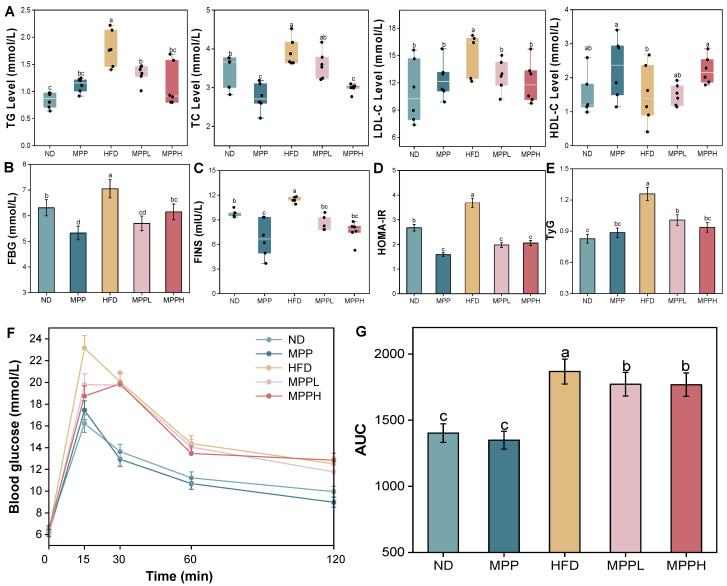
The effect of MPP on blood lipid levels and glucose and lipid metabolism in obese mice. Blood lipid level indicators: TC, TG, LDL-C, and HDL-C (**A**). FBG (**B**). FINS (**C**). HOMA-IR (**D**). TyG (**E**). OGTT (**F**). Area under OGTT experimental curve (**G**). ND, MPP, HFD, MPPL, and MPPH are the names of the five experimental groups in this study, representing, respectively, normal diet and saline solution, normal diet and MPP 200 mg/kg BW, high-fat diet and saline solution, high-fat diet and MPP 100 mg/kg BW, and high-fat diet and MPP 200 mg/kg BW. Data are mean ± SD (*n* = 6). Data with different letters indicate significantly differences (*p* < 0.05).

As shown in Figure 4, after a 9-week intervention, the serum levels of TC, TG, and LDL-C increased significantly and the serum levels of HDL-C levels decreased significantly in the HFD group. Excessive accumulation of TG in adipocytes is a key contributor to obesity [53]. In particular, the MPP treatment had a mitigating effect on lipid levels and was most effective in the high-dose (MPPH) group. Meanwhile, in the HFD group, compared with the ND group, fasting blood glucose was significantly elevated, while the MPP-treated group reduced fasting blood glucose levels (Figure 4B). In summary, MPP exerts modulatory effects on lipid and glucose metabolism in HFD-induced obese mice.

To assess MPP’s regulatory effects on glucose–lipid homeostasis in diet-induced obesity models, OGTT and ITT were systematically conducted. These standard metabolic evaluations provide dynamic quantification of β-cell function and insulin resistance, serving as cornerstone methodologies in metabolic syndrome research [54]. As shown in Figure 4F, the analysis of the blood glucose curves over 120 min following oral glucose administration revealed that the AUC in the HFD group was significantly increased compared to the other groups, while the AUCs in the MPPL and MPPH groups were significantly decreased and comparable to each other. In addition, fasting insulin levels were significantly higher in the HFD group than in the ND group (Figure 4C), and the HOMA-IR index was markedly elevated in the HFD group; MPP treatment significantly mitigated this elevation. To further validate these findings, the triglyceride–glucose index (TyG) exhibited trends congruent with HOMA-IR across all five groups (Figure 4D,E). These results demonstrate that MPP can modulate glucose homeostasis and insulin resistance in HFD-induced obese mice, suggesting a therapeutic potential for obesity intervention.

### 3.4. MPP Improved Gut Microbiota Dysbiosis in HFD-Induced Obese Mice

**Figure 5:**
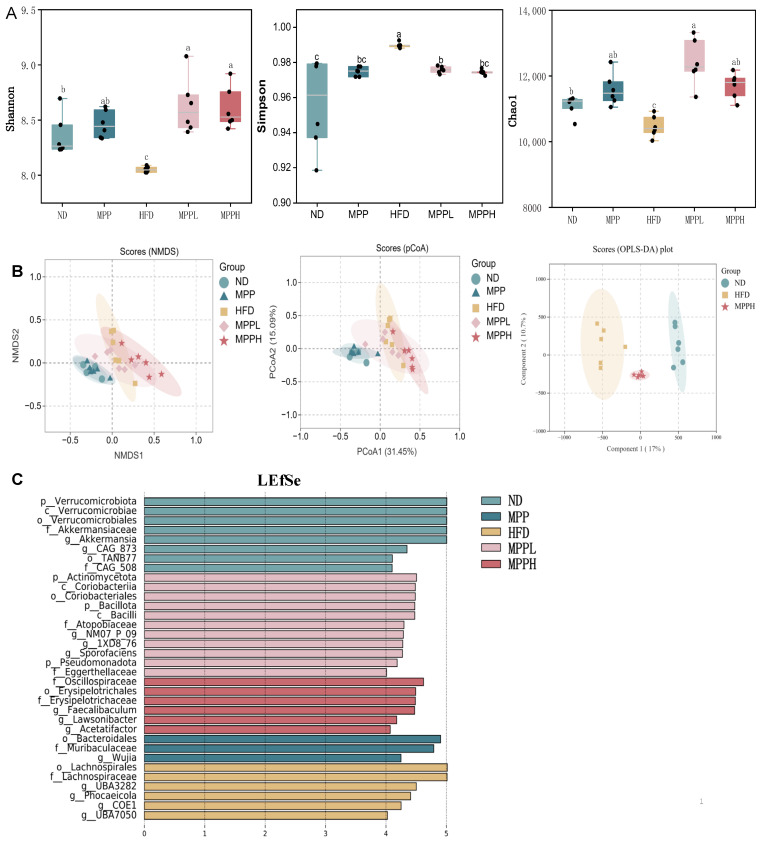
The effect of MPP on gut microbiota diversity in obese mice. Diversity indicators (**A**). Component analysis (**B**). LEfSe analysis (**C**). ND, MPP, HFD, MPPL, and MPPH are the names of the five experimental groups in this study, representing, respectively, normal diet and saline solution, normal diet and MPP 200 mg/kg BW, high-fat diet and saline solution, high-fat diet and MPP 100 mg/kg BW, and high-fat diet and MPP 200 mg/kg BW. Data are mean ± SD (*n* = 6). Data with different letters indicate significantly differences (*p* < 0.05).

Accumulating evidence positions gut microbiota dysbiosis as a critical determinant in obesity pathogenesis. Experimental models demonstrate that chronic high-fat dietary exposure not only drives metabolic syndrome development but also induces gut microbiota remodeling, creating a pathological feedback loop that perpetuates adiposity [55], building on previous research, and conducting joint analysis of multiple omics studies [56]. As shown in Figure 5A, we evaluated the diversity of gut microbiota by measuring the Shannon, Chao1, and Simpson indices. These three core indices exhibit distinct analytical characteristics: the Shannon index integrates species richness and distribution uniformity, the Simpson index quantifies community dominance by estimating the probability that two randomly selected individuals belong to the same species, and the Chao1 index statistically extrapolates the total number of species based on low-abundance taxa. Generally, higher Shannon index values indicate greater species diversity and uniformity, whereas higher Simpson index values reflect lower diversity due to the dominance. The results show the Shannon and Chao1 indices were decreased in the HFD groups compared to the ND group, while after the MPP treatment, the Shannon and Chao1 indices were significantly increased in MPPL and MPPH groups. Meanwhile, the Simpson index increased in the HFD group, while it was significantly decreased in the MPPL and MPPH groups. Previous studies have shown that obese mice have a lower diversity of gut microbiota than normal mice, which is consistent with our results [57]. This suggests that MPP can ameliorate obesity-induced reductions in species diversity. To further observe the effect of MPP on gut microbial diversity, pCoA, NMDS, and OPLS-DA analyses are presented in Figure 5B. The pCoA results show that the MPPH group was separated from the HFD group, indicating that high-dose MPP may reverse the changes in microbial community structure caused by a high-fat diet. The OPLS-DA results show that the MPPH group data were located between the ND and HFD groups, and both Q^2^ and *p* values were within the standard range, indicating that MPP restructures the obesity-related gut microbiota. These analyses indicated that MPP impacts bacterial community structure and alters microbial diversity in HFD-induced mice. Meanwhile, the LEfSe analysis also demonstrated significant alterations in gut microbiota among the five groups (Figure 5C).

**Figure 6:**
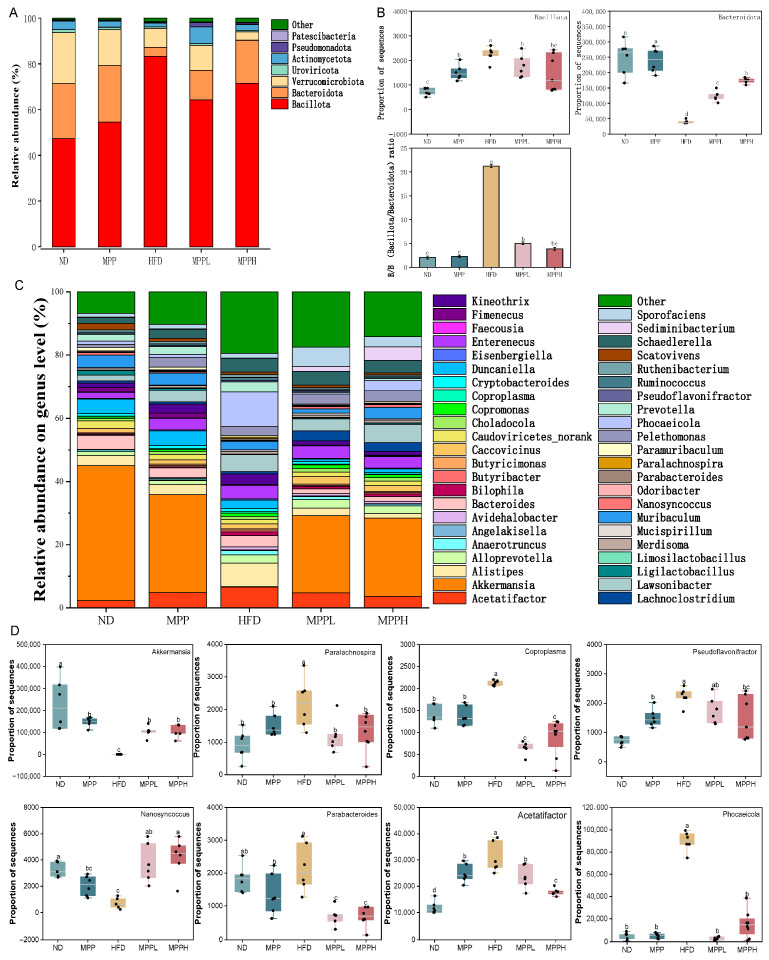
The effect of MPP on gut microbiota in obese mice. The effect of MPP at the phylum level of obese mice (**A**). Main microbial changes at the phylum level (**B**). The effect of MPP at the genus level of obese mice (**C**). Main microbial changes at the genus level (**D**). ND, MPP, HFD, MPPL, and MPPH are the names of the five experimental groups in this study, representing, respectively, normal diet and saline solution, normal diet and MPP 200 mg/kg BW, high- fat diet and saline solution, high-fat diet and MPP 100 mg/kg BW, and high-fat diet and MPP 200 mg/kg BW. Data are mean ± SD (*n* = 6). Data with different letters indicate significantly differences (*p* < 0.05).

At the phylum level, Bacillota and Bacteroidota were the two most dominant groups, while Verrucomicrobiota, Uroviricota, Pseudomonadota, Actinomycetota, and Patescibacteria comprised the remaining significant five phyla (Figure 6A). Among them, the abundance of Bacillota and Bacteroidota showed the most significant changes. The abundance of Bacillota increased significantly in the HFD group compared to the ND group. Following MPP treatment, however, the abundance of Bacillota decreased in a dose-dependent manner and returned to near-normal levels in both the MPPL and MPPH groups. In contrast, the abundance of Bacteroidota was significantly reduced in the HFD group compared to the ND group and increased dose-dependently after MPP intervention (Figure 6B). At the genus level, further changes in gut microbiology were observed (Figure 6C,D). Genera such as *Paralachnospira*, *Coproplasma*, *Pseudoflavonifractor*, *Parabacteroides*, *Acetatifactor*, and *Phocaeicola* were significantly increased in the HFD group compared to the ND group, but their abundances were significantly decreased in the MPPL group and the MPPH group. In contrast, the abundances of *Akkermansia* and *Nanosyncoccus*, which were significantly decreased in the HFD group, returned to normal levels in the MPPL and MPPH groups. In summary, MPP exerts microbiota-modulating effects through the bidirectional regulation of gut microbial ecology, while enhancing the proliferation of SCFA-producing taxa, thereby restoring the HFD-induced disruption of colonic microbial homeostasis.

### 3.5. MPP Regulates SCFAs in HFD-Induced Obese Mice

**Figure 7:**
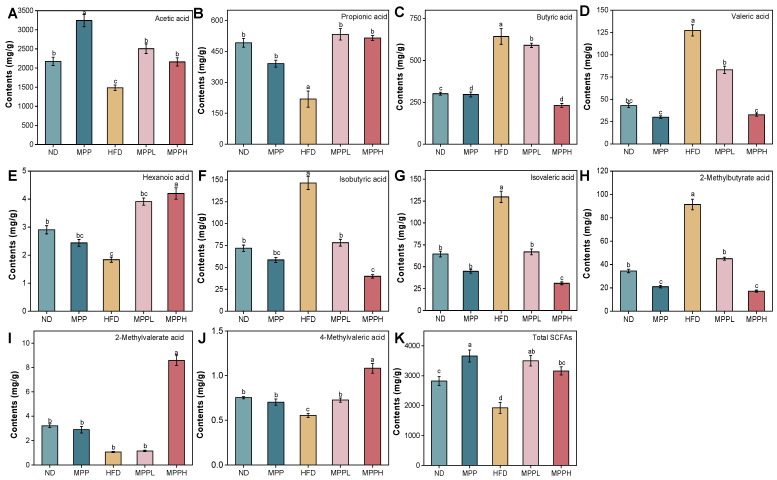
The effect of MPP on short-chain fatty acids in obese mice. Acetic acid (**A**). Propionic acid (**B**). Butyric acid (**C**). Valeric acid (**D**). Hexanoic acid (**E**). Isobutyric acid (**F**). Isovaleric acid (**G**). 2-Methylbutyrate acid (**H**). 2-Methylvalerate acid (**I**). 4-Methylvaleric acid (**J**). Total SCFAs (**K**). ND, MPP, HFD, MPPL, and MPPH are the names of the five experimental groups in this study, representing, respectively, normal diet and saline solution, normal diet and MPP 200 mg/kg BW, high-fat diet and saline solution, high-fat diet and MPP 100 mg/kg BW, and high-fat diet and MPP 200 mg/kg BW. Data are mean ± SD (*n* = 6). Data with different letters indicate significantly differences (*p* < 0.05).

Figure 7 demonstrates significant gut microbiota-derived SCFA dysregulation in the HFD model. SCFAs were measured in fecal samples from the colon of mice. Compared to the ND group, the HFD group exhibited significantly elevated concentrations of butyric acid, valeric acid, isobutyric acid, isovaleric acid, and 2-methylbutyric acid, while the MPP treatment dose-dependently reduced these SCFAs, with a significant reduction observed in the MPPH group. Comparative analysis revealed distinct variations in short-chain fatty acid profiles among the experimental groups. Relative to the ND group, the HFD cohort exhibited marked reductions in acetic acid, propionic acid, hexanoic acid, 2-methylvaleric acid, 4-methylvaleric acid concentrations, and total SCFA content decreasing. Conversely, the MPP intervention demonstrated significant restoration effects, elevating these specific fatty acids and total SCFA compared to the HFD controls. In addition, SCFAs enhance insulin sensitivity and reduce adiposity [58]. Further, monocarboxylate transporter 1 (MCT1) has been shown to be a major lactate transporter protein in adipocytes, and MCT1 is one of the important transport pathways for butyric acid [59]. As shown in Figure 7, MPP reduces butyric acid production in the gut, which may affect MCT1 function and ultimately lead to reduced lipid accumulation in adipocytes. The anti-obesity mechanisms of SCFAs include enhanced fatty acid oxidation in peripheral tissues and reduced lipid deposition in white adipose tissue [60].

In addition, the anti-obesity effects of MPP are associated with changes in SCFAs concentrations, which are closely linked to the gut microbiota that produce them. Bacteroidota primarily produce acetic acid and propionic acid, whereas Bacillota mainly produce butyric acid [61]. Research has shown that Bacteroidota and Bacillota are closely associated with obesity-related metabolic diseases [62]. Bacteroidota regulates immune balance and T regulatory-cell differentiation, thereby inhibiting an inflammatory response and keeping the gut microbiota stable and achieving the effect of reducing obesity [63]. On the other hand, inflammation can be suppressed by reducing butyric acid and the bacteria that produce butyric acid [64], and inflammation itself was closely linked to obesity [65]. The MPP treatment reduced the content of butyric acid and valeric acid, and increased the content of acetic acid and propionic acid; these changes were accompanied by modulation of the abundances of Bacteroidota and Bacillota. Additionally, MPP increased the concentrations of SCFAs, such as propionic acid and hexanoic acid. The correlation analysis revealed that the abundances of *Paralachnospira*, *Ruminococcus*, *Parabacteroides*, and *Coproplasma* were negatively correlated with propionic acid and hexanoic acid, whereas *Nanosyncoccus* was positively correlated with propionic acid. *Ruminococcus* has been confirmed to be a harmful bacterium that can exacerbate obesity in the body [66]. *Coproplasma* belongs to Bacillota, mainly producing butyric acid, and the MPP treatment significantly reduces it. *Parabacteroides* and *Phocaeicola* have the main characteristics of carbohydrate metabolism and secretion of SCFA, which are closely related to metabolic syndrome, obesity, and inflammation in the body. MPP alleviates obesity by reducing *Parabacteroides* abundance, which is in accordance with former investigations [67,68]. *Acetatifactor* is a bacterium closely related to inflammation [69], and MPP reduces *Acetatifactor* to alleviate obesity symptoms in HFD mice. It has been demonstrated that the HFD diet causes an increase in *Acetatifactor* abundance and that the MPP treatment just mitigates this increase and shows a dose-dependent effect [70]. However, there have been no reported studies on the association between *Nanosyncoccus* and obesity. Interestingly, after the MPP treatment, the abundance of *Akkermansia* significantly increased, which is consistent with previous studies [71]. *Akkermansia*, a mucin-degrading bacterium, improves glucose tolerance, insulin sensitivity, and intestinal mucosal integrity, thereby alleviating inflammation and obesity. It also produces propionic acid and exhibits anti-inflammatory and anti-obesity effects [72]. The experimental results validate this, with the MPP-treated group showing a significant increase in *Akkermansia* abundance. Collectively, MPP demonstrates anti-obesity efficacy through the bidirectional modulation of gut microbiota ecology and restoration of microbial metabolite homeostasis, particularly in normalizing SCFA biosynthesis pathways.

### 3.6. Correlation Analysis

**Figure 8:**
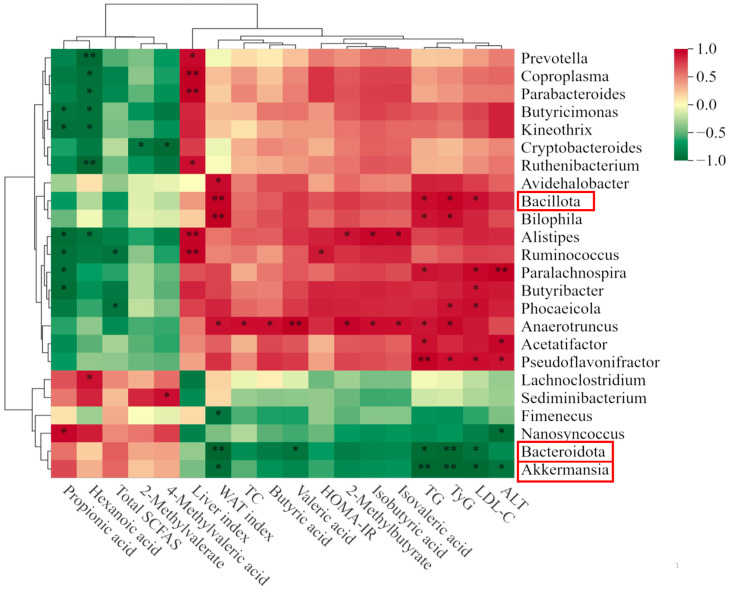
Correlation analysis between gut microbiota and physiological and biochemical indicators in obese mice (* *p* < 0.05, ** *p* < 0.01, the red wireframe indicates microorganisms that have a significant impact).

In order to further explore the association between gut microbiota and HFD-induced obese mice, a Spearman correlation heatmap analysis was performed, as shown in Figure 8. At the phylum level, Bacillota was positively correlated with TG, LDL-C, TyG (*p* < 0.05), and the WAT index (*p* < 0.01), whereas Bacteroidota was negatively correlated with LDL-C, TG (*p* < 0.05), and with both the WAT index and TyG (*p* < 0.01). At the genus level, *Akkermansia* was negatively correlated with the WAT index, ALT, LDL-C, TyG (*p* < 0.05), and TG (*p* < 0.01). In addition, *Phocaeicola* and *Ruminococcus* were negatively correlated with total SCFAs (*p* < 0.01). As shown in Figure 4, MPP exhibited hypolipidemic and hypoglycemic effects on HFD-induced obese mice. The reduction in FBG, TC, TG, and LDL-C may be related to the decreased size and improved function of adipocytes, which further explains the improvement in glucose tolerance observed in HFD-induced obese mice treated with MPP. Moreover, Bacillota and *Pseudoflavonifractor* were positively correlated with TC, TG, and LDL-C, suggesting that the reduction in these lipids may be attributed to the inhibition of Bacillota and *Pseudoflavonifractor* production by MPP, which is consistent with previous studies [73]. It is worth noting that this study has some limitations. Firstly, only an obese mouse model was employed, and while the results demonstrate that MPP effectively improves HFD-induced obesity in mice, its efficacy remains unverified in other models or humans. Secondly, compared to commercial pectin, the MPP extracted and purified in this study exhibits limitations such as low purification levels. Therefore, this study primarily demonstrates that MPP alleviates HFD-induced obesity symptoms, providing foundational insights and a theoretical basis for mango peel utilization.

## 4. Conclusions

This study characterizes mango peel pectin (MPP) with a molecular weight of 6.76 × 10^5^ Da and monosaccharide composition dominated by galacturonic acid (21.36%), glucose (8.85%), and arabinose (5.97%). MPP significantly attenuated high-fat diet (HFD)-induced weight gain, ameliorated hepatic steatosis and adipocyte hypertrophy, and reduced systemic lipid accumulation. Furthermore, MPP improved glucose–lipid metabolic parameters, enhanced glucose tolerance, and restored insulin sensitivity. Mechanistically, MPP normalized dysregulated glucolipid homeostasis by reinforcing insulin signaling pathways and modulating the enterohepatic axis via microbial-derived metabolites. These effects corresponded with specific gut microbiota remodeling: increased abundance of beneficial taxa (e.g., Bacteroidota, *Akkermansia*, *Nanosynococcus*) and decreased proliferation of obesity-associated genera (Bacillota, *Coproplasma*, *Parabacteroides*, *Acetatifactor*, *Phocaeicola*). Collectively, these findings establish MPP as a functionally active dietary fiber and provide foundational data for valorizing mango peel in nutraceutical applications.

## Figures and Tables

**Table 1:** Methylation analysis data of MPP.

Linkage Types	Methylated Sugar	Mass Fragments (*m/z*)	Molar Ratio
t-Ara(*f*)	1,4-di-*O*-acetyl-2,3,5-tri-*O*-methyl arabinitol	279	9.92
t-Glc(*p*)	1,5-di-*O*-acetyl-2,3,4,6-tetra-*O*-methyl glucitol	323	3.43
t-Gal(*p*)-UA	1,5-di-*O*-acetyl-2,3,4,6-tetra-*O*-methyl galactitol	325	8.93
5-Ara(*f*)	1,4,5-tri-*O*-acetyl-2,3-di-*O*-methyl arabinitol	307	5.18
4-Gal(*p*)-UA	1,4,5-tri-*O*-acetyl-2,3,6-tri-*O*-methyl galactitol	353	32.13
4-Gal(*p*)	1,4,5-tri-*O*-acetyl-2,3,6-tri-*O*-methyl galactitol	351	8.08
4-Glc(*p*)	1,4,5-tri-*O*-acetyl-2,3,6-tri-*O*-methyl glucitol	351	22.24
6-Gal(*p*)	1,5,6-tri-*O*-acetyl-2,3,4-tri-*O*-methyl galactitol	351	4.12
2,4-Glc(*p*)	1,2,4,5-tetra-*O*-acetyl-3,6-di-*O*-methyl glucitol	379	1.41
4,6-Gal(*p*)	1,4,5,6-tetra-*O*-acetyl-2,3-di-*O*-methyl galactitol	379	1.65
3,6-Gal(*p*)	1,3,5,6-tetra-*O*-acetyl-2,4-di-*O*-methyl galactitol	379	2.90

**Table 2:** ^1^H and ^13^C NMR chemical shifts (ppm) of MPP.

Glycosyl Residues		Chemical Shifts (ppm)					
		H1/C1	H2/C2	H3/C3	H4/C4	H5/C5	H6/C6
A	→4)-α-D-GalpA-OMe-(1→	5.34	3.57	3.78	3.89	3.87	/
		99.7	71.47	71.15	76.58	73.28	170.67
B	→4)-β-D-Glcp-(1→	4.42	3.48	3.86	3.61	3.87	3.66, 3.76
		103.41	70.87	69.44	76.76	73.63	61.07
C	→4)-β-D-Galp-(1→	4.58	3.62	3.98	4.22	n.d	3.78, 3.6
		104.26	72.71	69.46	79.35	n.d	60.47
D	→6)-α-D-Galp-(1→	5.09	4.05	n.d	n.d	n.d	3.74, 3.83
		107.06	80.87	n.d	n.d	n.d	66.32
E	→5)-α-L-Araf-(1→	5.03	4.09	n.d	4.15	3.89	/
		107.46	81.08	n.d	82.18	66.51	/
F	α-L-Araf-(1→	5.18	4.11	n.d	4.25	3.43	/
		109.31	77.75	n.d	81.42	60.04	/
G	→3,6)-α-D-Galp-(1→	4.9	3.66	3.98	n.d	n.d	3.91,4.07
		100.6	74.82	76.66	n.d	n.d	68.46

## Data Availability

The original contributions presented in the study are included in the article. Further inquiries can be directed to the corresponding author.

## Supplementary Materials

The following are available online at https://www.mdpi.com/article/10.3390/foods14162910/s1. Table S1, Effects of MPP supplementation on fasting body weight, FBG, food intake, water consumption and organ index in mice.

## Abbreviations

The following abbreviations are used in this manuscript:

WHO	World Health Organization
SCFAs	Short-chain fatty acids
MPP	Mango peel pectin
TG	Triglyceride
TC	Total cholesterol
LDL-C	Low-density lipoprotein cholesterol
HDL-C	High-density lipoprotein cholesterol
AST	Aspartate transaminase
ALT	Alanine aminotransferase
Mw	Molecular weight
HPGPC	High-performance gel-permeation chromatography
FTIR	Fourier Transform Infrared
GC-MS	Gas chromatography-mass spectrometry
DCM	Dichloromethane
NMR	Nuclear magnetic resonance
COSY	Correlation spectroscopy
HSQC	Heteronuclear single-quantum coherence
HMBC	Heteronuclear multiple-bond correlation
NOESY	Nuclear Overhauser effect spectroscopy
SPF	Specific pathogen free
ND	Normal diet
HFD	High-fat diet
BW	Body weight
WAT	White adipose tissue
H&E	Hematoxylin and eosin
OGTT	Oral glucose tolerance test
SD	Standard deviation
AUC	Area under the curve
HOMA-IR	Homeostasis model assessment
FBG	Fasting blood glucose
PCA	Principal component analysis
DM	Dispersity
RG-I	Rhamenogalacturonan-I
RG-II	Rhamenogalacturonan-II
D-GalA	D-galacturonic acid
D-Gal	D-galactose
D-Glu	D-glucose
l-Ara	L-arabinose
TyG	Triglyceride–glucose index
ANOVA	Analysis of variance
pCOA	Principal co-ordinate analysis
LefSe	Linear discriminant analysis effect size
OPLS-DA	Orthogonal projections to latent structures discriminant analysis
NMDS	Nonmetric multidimensional scaling
