# Supplementary material for: Structural Characterization of a Novel Pectin Polysaccharide from Mango (Mangifera indica L.) Peel and Its Regulatory Effects on the Gut Microbiota in High-Fat Diet-Induced Obese Mice
Source: Foods. 2025 Aug 21;14(16):2910. doi: 10.3390/foods14162910 (PMC12385833; doi:10.3390/foods14162910)
Supplement: Supplementary file 1 [file foods-14-02910-s001.zip › foods-3764180-supplementary.pdf]

Table S1. Effects of MPP supplementation on fasting body weight, FBG, food intake, water consumption and organ index in mice.

		ND	MPP	HFD	MPPL	MPPH
Body weight(g)	0 week	22.35±0.25ab	21.74±0.30b	22.89±0.32a	22.07±0.15ab	22.37±0.30ab
	1 week	23.43±0.27cd	22.98±0.42d	26.04±0.34a	24.40±0.19bc	24.62±0.5b
	2 weeks	25.24±0.50b	23.73±0.48c	27.98±0.42a	25.71±0.32b	25.72±0.54b
	3 weeks	26.37±0.56b	24.02±0.48c	28.96±0.51a	26.70±0.40b	26.42±0.57b
	4 weeks	27.56±0.62b	25.23±0.61c	31.45±0.55a	28.47±0.47b	27.98±0.70b
	5 weeks	28.38±0.60b	25.73±0.55c	33.04±0.66a	30.06±0.61b	29.19±0.77b
	6 weeks	28.81±0.63c	26.15±0.58d	33.26±0.59a	31.06±0.69b	30.15±0.81bc
	7weeks	29.36±0.72c	26.45±0.53d	35.75±0.78a	32.23±0.78b	30.77±0.84bc
	8 weeks	30.48±0.74c	27.38±0.55d	37.76±0.84a	34.16±0.92b	32.08±0.88bc
	9 weeks	30.89±0.87c	27.56±0.58d	39.43±1.05a	35.65±1.10b	33.40±0.93bc
Body weight alteration (g)		+8.54c	+5.82d	+16.54a	+13.58b	+11.03bc
Food intake (g/mouse/day)	1 week	3.28±0.15a	3.06±0.04a	2.58±0.12b	2.35±0.05b	2.37±0.084b
	2 week	3.21±0.31a	2.79±0.38ab	2.60±0.08b	2.28±0.02b	2.26±0.26b
	3 weeks	3.46±0.25a	2.94±0.04b	3.43±0.01a	3.00±0.04b	2.83±0.12b
	4 weeks	3.42±0.32a	2.80±0.05b	3.73±0.09a	3.28±0.03ab	3.26±0.02ab
	5 weeks	3.79±0.50a	3.12±0.15a	3.12±0.42a	3.22±0.04a	2.93±0.08a
	6 weeks	3.74±0.29a	3.21±0.03abc	3.44±0.19ab	2.98±0.01bc	2.77±0.08c
	7 weeks	3.99±0.41a	3.30±0.03ab	3.40±0.09ab	3.18±0.04b	2.67±0.14b
	8 weeks	4.28±0.60ab	4.68±0.01a	3.80±0.04abc	3.45±0.06bc	3.03±0.17c
Water consumption (mL/mouse/day)	1 week	4.54±0.11a	4.33±0.14ab	3.82±0.45ab	3.70±0.06b	3.58±0.41b
	2 week	5.10±0.60a	4.25±0.21ab	4.14±0.38ab	4.09±0.26ab	3.42±0.03b
	3 weeks	5.29±0.52a	4.26±0.22ab	4.26±0.44ab	4.02±0.11ab	3.62±0.48b
	4 weeks	5.60±0.63a	4.58±0.36ab	4.55±0.06ab	4.36±0.13ab	3.99±0.08b
	5 weeks	5.07±0.48a	4.34±0.38a	4.44±0.21a	3.58±0.53a	3.75±0.28a
	6 weeks	5.47±0.67a	4.48±0.23ab	4.37±0.07ab	4.09±0.14ab	3.89±0.44b
	7 weeks	5.81±0.59a	4.78±0.10ab	4.37±0.03b	4.24±0.26b	3.78±0.33b

	8 weeks	6.12±0.90a	4.55±0.34ab	4.15±0.06b	4.16±0.06b	3.82±0.21b
Organ index (%)	Liver	3.17±0.18b	3.14±0.27b	3.71±0.23a	2.65±0.10c	2.62±013c
	WAT	1.75±0.20c	1.40±0.19c	6.98±0.98a	4.29±0.27b	4.21±0.73b
